# Comprehensive Analysis of Network Slicing for the Developing Commercial Needs and Networking Challenges

**DOI:** 10.3390/s22176623

**Published:** 2022-09-01

**Authors:** Sumbal Zahoor, Ishtiaq Ahmad, Mohamed Tahar Ben Othman, Ali Mamoon, Ateeq Ur Rehman, Muhammad Shafiq, Habib Hamam

**Affiliations:** 1 Department of Electrical Engineering, The University of Lahore, Lahore 54000, Pakistan; 2 Department of Computer Science, College of Computer, Qassim University, Buraydah 51452, Saudi Arabia; 3 Department of Software Engineering, Calrom, Manchester M1 6EG, UK; 4 Department of Electrical Engineering, Government College University, Lahore 54000, Pakistan; 5 Department of Information and Communication Engineering, Yeungnam University, Gyeongsan 38541, Korea; 6 Faculty of Engineering, Uni de Moncton, Moncton, NB E1A 3E9, Canada; 7 International Institute of Technology and Management, Libreville BP1989, Gabon; 8 Spectrum of Knowledge Production and Skills Development, Sfax 3027, Tunisia; 9 Department of Electrical and Electronic Engineering Science, School of Electrical Engineering, University of Johannesburg, Johannesburg 2006, South Africa

**Keywords:** network slicing, CSPs, automation, orchestration, code flow, NS projects, AI, ML

## Abstract

Network slicing (NS) is one of the most prominent next-generation wireless cellular technology use cases, promising to unlock the core benefits of 5G network architecture by allowing communication service providers (CSPs) and operators to construct scalable and customized logical networks. This, in turn, enables telcos to reach the full potential of their infrastructure by offering customers tailored networking solutions that meet their specific needs, which is critical in an era where no two businesses have the same requirements. This article presents a commercial overview of NS, as well as the need for a slicing automation and orchestration framework. Furthermore, it will address the current NS project objectives along with the complex functional execution of NS code flow. A summary of activities in important standards development groups and industrial forums relevant to artificial intelligence (AI) and machine learning (ML) is also provided. Finally, we identify various open research problems and potential answers to provide future guidance.

## 1. Introduction

The fifth generation (5G) with its next-generation wireless network (NGWN) architecture, is expected to undergo a significant transition to accommodate the billions of devices that will be linked to the internet. The development of an NGWN is highly anticipated as it is one of the world’s fastest and most resilient technologies that are capable of faster speeds, reduced latency, and enormous capacity. Another important distinction of 5G is its (NS) capabilities, which enable unique network traffic partitioned or sliced to meet the needs of diverse applications [1]. Although NS offers on-demand services, many of its applications would need the adoption of a multi-access edge computing (MEC) stage in 5G networks. In other words, 5G enables data to split like a pie, with one slice dedicated to low-latency applications such as transportation infrastructure, another to conventional consumer internet use, and another to machine-to-machine Internet of Things (IoT) services. According to the third-generation partnership project (3GPP), NS is important for CSPs in terms of developing new services and business models. CSPs may utilize NS to generate more virtual slices to accommodate massive traffic increases and specialized user requirements [2]. As a result of this progression, experts from various standard development organizations (SDOs) established a multi-vendor landscape that creates NS standards and recommendations [3]. With the appropriate strategy, telcos can optimize the network to effectively give quality of service (QoS) as a service while supporting diverse use cases such as real-time analytics via edge computing, remote operations via augmented reality (AR) technologies, AI-enabled autonomous cars, and real-time video monitoring of remote manufacturing plants [4].

**Figure 1:**
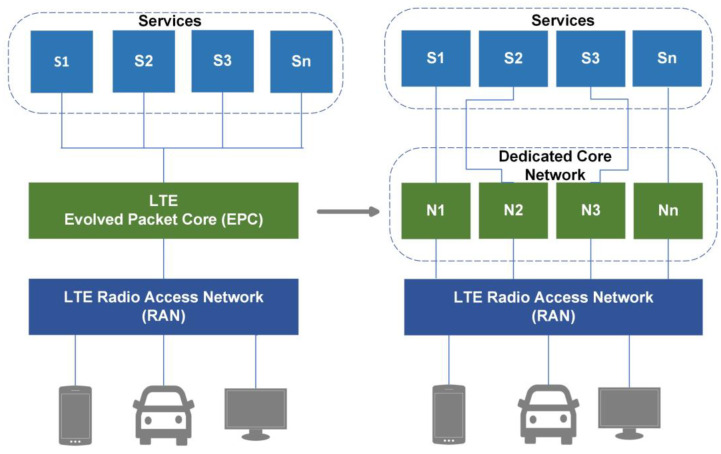
Evolution of NGWNs (multiple services sharing one link to 4G framework for dedicated core selection based on user equipment (UE)).

The transition to a new 5G core varies from previous mobile network generations because, with the advent of novel and disruptive networking paradigms, the conventional one-size-fits-all approach to network architecture is no longer suitable. Thus, the key ingredient for realizing the potential of 5G architecture is NS [5]. The idea of NS is not new since mobile broadband networks have always been successful in offering services to end-users by partitioning the network via access point names (APNs). Before the fourth generation (4G), a pre-3GPP release thirteen, the trend was to use handsets and the evolved-UMTS terrestrial radio access network (EUTRAN), which is a radio component of 4G (i.e., LTE RAN), as well as the evolved packet core (EPC) network. However, in 4G architecture, the concept of dedicated core networks (DCNs) also known as decor, is a signified evolutionary step in which the 3GPP built a framework that permits a DCN, or the separation of the main network into various DCNs that may be assigned to different clients. Figure 1 demonstrates how the transition from a single network offering all services to multiple DCN instances serving more targeted segments looks like [6].

### 1.1. Significance of Network Slicing

NS allows practical segments to be shared throughout distinct network slices while isolating each network slice and, as a result, keeping each slice at a safe distance from incursion. Each network slice may therefore be independently supervised and managed. It utilizes the protocols underlying software-defined networking (SDN) and network function virtualization (NFV) and works on virtual system engineering. Depending on the kind of RAN, administration, or geographic location, NS gives operators the authority to categorize certain system components. Operators might choose capacity, security, latency, network (fixed or WiFi), inclusion, and service level agreement (SLA) procedures for each slice. Respectively, this opens the chance to grow the client base and offer new, distinguished services.

The Global System for Mobile Communications Association (GSMA) approximates that there will be around 1.2 billion 5G networks by 2025, accounting for 40% of the worldwide inhabitants, or around 2.7 billion persons. It assumes that the future 5G network design is “a real break to produce a responsive network that adjusts to the diverse requirements of particular trades and the budget.” It is a vital feature of 5G networks utilized to recover the distribution of assets and increase cost and energy efficiencies. 

### 1.2. Fifth-Generation Use Cases

**Figure 2:**
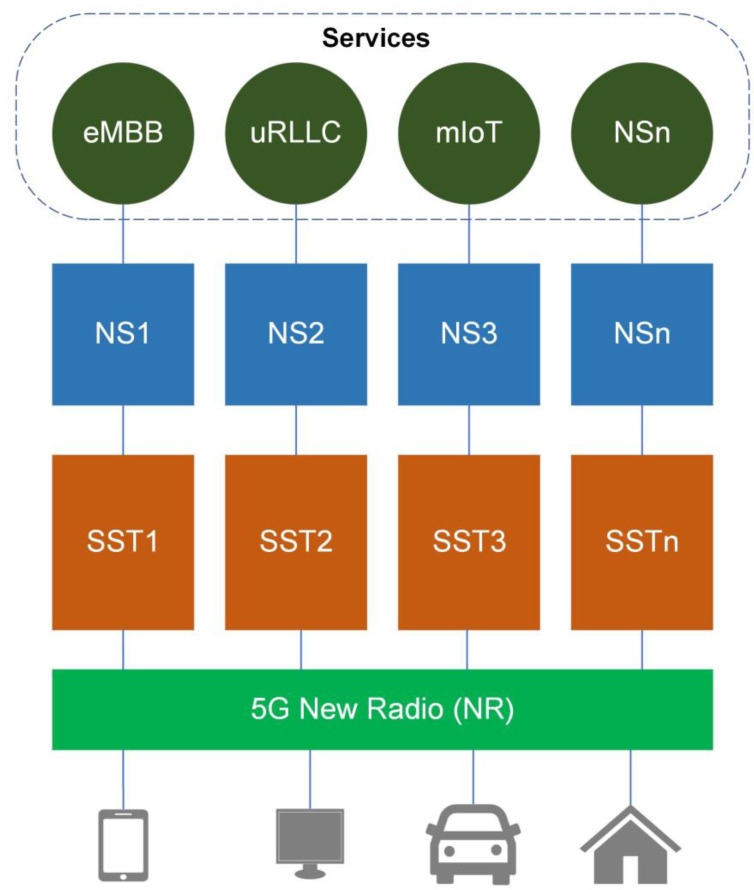
The 5G framework for NS based on SST.

A common framework called network slice instance (NSI), which theoretically splits the network into various slices, is designed to support 5G NS using the 4G dedicated core logic. To give more flexibility for dynamic slices addressing multiple marketing groups, the international telecommunication union (ITU) has grouped the 5G versatile system into three classes especially used for global interoperability enhanced mobile broadband (eMBB), ultra-reliable low latency communication (uRLLC), and massive Internet of Things (mIoT) [7]. These slices are universally acknowledged by slice/service type (SST), which reflects the anticipated network behavior in terms of features and services as presented in Figure 2.

As listed in Table 1, the 3GPP defines three standardized SSTs [8].

### 1.3. Network Slicing Classifications

The concept of NS can be extensively ordered into two classes: horizontal network slicing (HNS) and vertical network slicing (VNS). HNS permits asset allocation among various system nodes to improve the capacities of less competent system nodes. Hence, this approach requires over-the-air asset distribution through network nodes. In this technique, novel capacities can be included for a system node once assisting a particular system node. This method isolates registering assets, giving limit scaling, edge processing, and offloading. With an HNS, the end-to-end (E2E) traffic travels locally between the central system and the end-user [9]. 

VNS permits asset sharing between a variety of applications and services to improve the QoS. In VNS, every node of the network actualizes comparative capacities inside a particular slice. This type of slicing will isolate traffic for every application premise, giving clients on-request transfer speed. With a VNS, the E2E traffic typically travels between the central system and the end-user [10]. Irrespective of how network slices are categorized, one main takeaway from a VNS vs. HNS debate is the statistic that NS systems are going to have to see a variety of demands targeted at various businesses. This evidently points to the requirement for dynamic, on request NS proficiencies, as a primary phase, network expertise providers must study first rolling-out pre-defined, generic network slices intended for vertical and horizontal use cases.

### 1.4. Major Contributions and Paper Organization

To the best of our knowledge, we are the first to perform systematic research to explore the technology from a different perspective and to adopt a rational approach with the relevance of standardization bodies in the open-source communities to bring content that creates a practical sense. 

Several studies [11,12,13,14,15] presented a comprehensive investigation of NS. In [11], Foukas et al. offered an enhanced NS paradigm and evaluated it considering the evolution of previous concepts, along with outlining open research issues. Afolabi et al. [12] conducted a comprehensive evaluation of NS management and orchestration, functional prototypes, and supporting technologies. In addition, a set of criteria for the CN and RAN are specified to enable NS. The authors of [13] discussed the motivations and key enabling technologies, such as dynamic service chaining, governance, and coordination. The most recent advancements in 3GPP standards and 5G NS industry implementation were also discussed, as well as a few key outstanding concerns and obstacles. The use of NS and various settings to enable smart services in future networks is explored in [14]. This survey [15] investigated the use of NS on the Internet of Things. They also discussed the technological difficulties that NS can address, as well as the significance of emerging technologies and concepts such as blockchain and AI/ML.

Table 2 compares our survey contributions to several existing studies, indicating our originality in relation to past investigations. The following are the key points raised in this paper:

- Highlight 5G orchestration architecture, and the applicability of cloud-native supporting technologies.
- A thorough explanation of the significance of NS characterization is needed to design complete NS solutions. Additionally, we present a description of the technical and functional purpose of NS.
- Recent efforts are being made to support prospects for service providers.
- Provides a view into the AI- and ML-related activity taken on in various SDOs and commercial forums, and finally discussed the open research challenges faced by CSPs.

The following is how this article is arranged. Section 1 presents a high-level overview of NGWN development. Section 2 provides a business-oriented perspective of slicing in terms of prospects and predicted revenues, as well as an introduction to the network slice lifecycle management process. In Section 3, we discuss the requirement for a slicing automation and orchestration framework. Section 4 serves as a foundation for laying the essential NS elements of operators, frameworks, operations, and processes. Section 5 demonstrates the importance of AI and ML approaches for NS. Section 6 also shows unresolved research questions and responses to give future guidance. Section 7 served as the article’s conclusion. The definitions of widely used acronyms are shown in Table 3.

## 2. Network Slicing from a Business Standpoint

NS is one key technology that tells 5G apart from 4G, where a slice itself forms a logical private network, each optimized and designed for a specific purpose such as agricultural, mining, or vehicle-to-everything (V2X), depending on how CSP approaches the commercial market. Table 4 depicts enterprise requirements defining business product qualities [16].

According to a TM Forum poll, 71.5% of CSPs’ income will come from the enterprise business unit, but the challenge is to find the appropriate infographic. A survey by Capgemini showed that one-third of the detail organizations are considering obtaining their own 5G license, posing a threat to CSPs who will be left with no options. Slicing is linked with enterprise strategy, resulting in either initiative to remove operators from the equation by having a personal license and an E2E 5G solution or mobile operators maintaining a slot through NS [17]. 

**Figure 3:**
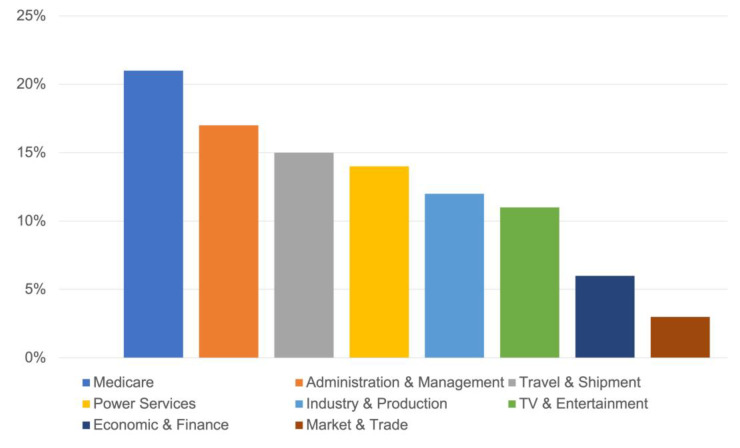
The view of end-user NS market.

The primary advantage of 5G for CSPs lies in the prospect to capture new profits in services for industries and enterprises. CSPs with NS have a USD 200 billion market potential. When broken down by industry, the top six industries account for 90% of the revenue potential. The major industry is healthcare, followed by government and transportation. The compound annual growth rate (CAGR) for the top industries varies from 23% to 46% between 2025 and 2030, which is significant as shown in Figure 3. Similarly, the researchers found that the NS industry is expected to grow in value from its anticipated value of USD 143.63 billion in 2020 to USD 446.33 billion by 2026, with a CAGR of 20.8 percent between 2021 and 2026. The expansion of this sub-segment in the worldwide NS market is mostly due to government support for the development of 5G infrastructure in the telecom [18].

### 2.1. Limitations and Challenges for Businesses Adopting 5G Technology

While 5G technology has advanced in recent years, it still has drawbacks, such as shorter service ranges and a limitation of remote deployments. However, the challenge or threat in adopting 5G in general or direct slicing is the lack of a business case since NS can be a vast number of slices, unlike in the past, requiring large spending on infrastructure automation and orchestration as it is a complete E2E system. Therefore, it is necessary to build the case and communicate with the European broadcasting union (EBU) with a clear pipeline of customers before investing; otherwise, it will pose a substantial risk [19]. Some of the major barriers to industries embracing 5G technology are as follows:

- **5G—A virtual adaptable network**
  The vast majority of 5G “non-standalone” installations presently rely on the existing 4G infrastructure. However, the industry is building a new network concept to realize the promise of 5G. The 5G core will be a “cloud-native”, Its underlying technologies SDN and NFV will virtualize basic network components. Furthermore, virtualization introduces new security issues since when a network is implemented in software, there is a risk of cross-contamination and data leakage. Automation can accelerate the spread of inaccurate judgments and illnesses.
- **Digitalization of customer connections**
  Telcos must investigate virtual alternatives that deliver attractive services to clients who are not there in person. Data are crucial in any situation. A mobile network operator (MNOs) should be able to examine digital interactions with the network to help comprehend individual subscribers’ behavior. With this information, businesses can give customized digital services.
- **Large-scale deployments of linked products are now feasible**
  The bulk of IoT devices are inexpensive, light, have little computational capability, and are powered by batteries. They should last for an extended period under challenging circumstances. The diversity of data connections is an additional factor. Some devices require low-bandwidth communication over short distances. Others demand sending brief, high-bandwidth bursts over considerable distances [20].
- **Businesses acquire communications freedom**
  Businesses will be adept to manage their private network slices in a 5G future. However, running an in-house network requires security knowledge since it creates sensitive data that must be protected both in transit and at rest. To assist, telecommunications companies must ensure that only authorized persons have access to this information. They can collaborate with these businesses as “security as a service” partners. Previously, MNOs concentrated on enhancing smartphone security. They will need to enhance their skills across different device kinds and industrial sectors in the 5G future.
- **Information structure must be cybersecure**
  The 5G network is the first mobile technology to launch in the era of global cybercrime conducted by professional syndicates. The virtual structure of the 5G network core provides these attackers with additional access opportunities. Because of virtualization, data no longer kept centrally but at the “edge." The 5G network also dramatically increases the number of linked devices.

### 2.2. Network Slice Lifecycle Management

**Figure 4:**
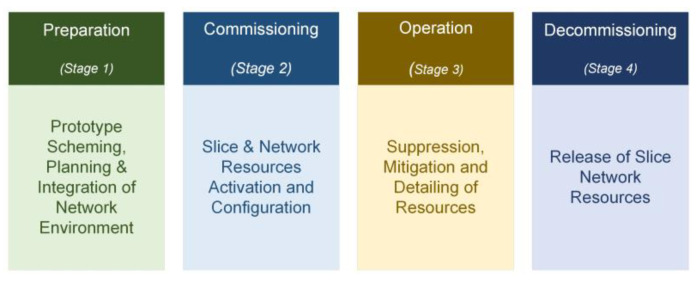
Organization of NS lifecycle.

When estimating business cases for different verticals with varying needs, it is necessary to create distinct products that can activate on-demand, since it is a very dynamic framework that cannot be achieved in previous solutions. Slice modeling is required to have templates accessible to fulfill vertical needs and to do on-demand instantiation based on client requirements [21]. The four phases depicted in Figure 4 outline the management features of a network slice instance.

- **Preparation:** this stage involves creating a network slice template, planning network slice capacity, onboarding, evaluating access network requirements, configuring the communication network, and performing any other prerequisite preparations before beginning an NSI.
- **Commissioning:** building the NSI is a part of provision at the assigned level. All essential responsibilities are scheduled and adjusted to fit the network slice criteria whenever an NSI is generated. The development and/or change of NSI components may be completed as part of the creation process.
- **Operation:** this phase comprises the initiation, management, performance reporting, improve resource allocation, customization, and termination of an NSI throughout the operation phase.
- **Decommissioning:** includes deactivating non-shared constituents as needed, as well as removing NSI-specific settings from common constituents. After the decommissioning procedure, the NSI is terminated and no longer exists.

Therefore, the lifecycle management technique demands constant monitoring, healing, and scaling, which can then export to the enterprise client to evaluate what is going on in that slice. Since it is a complete cycle of governance, the same framework is necessary to manage decommissioning. 

## 3. Fifth-Generation Orchestration Framework

The 5G network is intended to be service-oriented, with the idea that it will support a diverse set of services with varying configuration needs, such as automation, scalability, accounting, and network status monitoring. From a standard perspective, the number of slices can grow exponentially high, i.e., any CSP can govern 2^232^ slices, resulting in a substantial number of slices. To support this concept, 3GPP agreed in release 15 to adopt a service-based management architecture framework with the ability to interact with other architectural frameworks to jointly meet 3GPP service and network management expectations [22]. 

The capacity of networks to provide network slice as a service (NSaaS) that may license to organizations with QoS demands and capabilities has managed to expand the concept of NSaaS model. This approach enables a network service customer to order and configure a network slice, which then delivered as a service. To ensure that the specified SLA for the provided network slice is satisfied, monitoring and telemetry methods must be enabled at various resource levels, including the reconfigurable infrastructure, the 5G core network (CN), transport network (TN), and radio access network (RAN), and the NS resources. 

The term “communication service instance” (CSI) created by 3GPP describes a service provided to an end-user. Underneath, the CSI is mapped to a collection of network NSIs and the relevant resources (e.g., computational, memory, and connectivity) that form an ordered network slice, such that any source offered to an external client is labeled as a resource providing service to an NSI. Whereas the NSI is made up of multiple network slice subnets instances (NSSI), it is a representation of the management elements of a group of controlled functions and the required resources (e.g., computational, memory, and connectivity). Hence, according to the standards, these class subnets encompass three domains called RAN, TN, and CN [23].

In a nutshell, the new paradigm needs the development of an orchestration framework that manages network slice life cycles and provides domain orchestration capabilities. Some additional functional blocks included with 3GPP to indicate the role required to address the slicing are as follows [24]:

- **Communication Service Management Function (CSMF):** manages to transform communication service needs into network slice demands.
- **Network Slice Management Function (NSMF):** manages network slice templates (NSTs) including lifecycle management. The network slice subnet parameters are inferred from the network slice specifications.
- **Network Slice Subnet Management Function (NSSMF):** responsible for the management of NSIs
- **NF Management Function (NFMF):** responsible for application-level managing of virtual network functions (VNFs) and physical network functions (PNFs) and is a manufacturer of the provisioning solution that offers configuration management (CM), fault management (FM), and performance management (PM) [25].

In general, any CSP’s multi-domain orchestration and an E2E service orchestration layer will include CSMF/NSMF. The NSSMF includes slice intelligence; it takes an NSMF command, such as “create a slice,” and initiates it by performing all the work of role allocation, storage, installation, and consultation. 

### 3.1. Integrating 5G Orchestration with ETSI NFV

**Figure 5:**
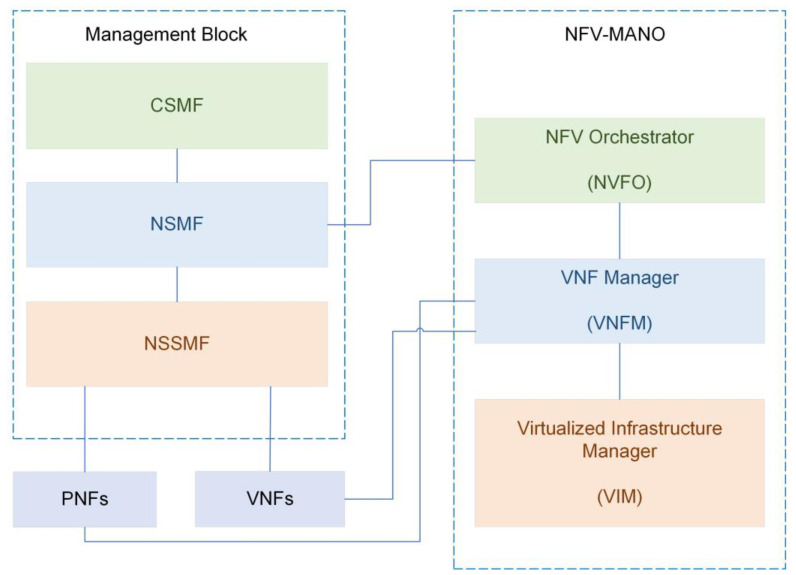
Fifth-generation orchestration with ETSI NFV.

This section provides interesting information from a 3GPP perspective to highlight how the management blocks are connected to the existing NFV stack. The management and orchestration (MANO) framework, represented on the right side of Figure 5, is made up of three primary construction blocks:

- The NFV orchestrator (NFVO) oversees orchestrating and managing the NS installed on the network functions virtualization infrastructure (NFVIs).
- The VNF manager directs the life-cycle management (LCM) of one or more VNFs. It is possible to deploy multiple VNF managers.
- The virtualized infrastructure manager (VIM) administers coordinating the functions needed to regulate and manage a VNF’s interface with computing, storage, and network resources, as well as their virtualization. Multiple VIM instances, one for each type of NFVI technology, might be implemented.

The NSMF is a domain orchestrator responsible to manage the slices in multiple domains and integrates to NSSMF in the core, access, and transport as well. It might function as a northbound system to the NFVO while the NFMF continues to perform its normal functions, such as integrating with VNFs and PNFs [26].

### 3.2. Cloud-Native Powering the Communication Industry’s Digital Transformation

To meet the diverse and changing demands of various sectors, the 5G slicing solution must be implemented rapidly on request, using cloud-native (next-generation virtualized CN architecture) concepts and models, which will surely service thousands of 5G industrial connections requirements for the digital revolution. The cloud-native core network (CNCF) provides telecom networks to better accommodate vertical sectors and assist industrial clients in accelerating digital transformation. Regardless of infrastructure, the CNCF is critical to meeting the cloud-native application micro-service component selection, grey upgrading, and flexible deployment needs. Many factors influence cloud-native’s speed and agility. To manage the complexity of 5G apps and service-based use cases, the orchestration required to manage cloud-native applications and infrastructure must be deployed and established.

### 3.3. Cloud-Native Empowering Technologies for NS

- **Containers**
  When it comes to standalone 5G networks, a variety of the world’s top mobile carriers are investigating a cloud-native network design combined with containers (lightweight virtualization alternative to virtual machines (VMs). Containers, as opposed to virtual machines, can reduce costs by packaging only the OS needs particular to a certain application. Docker is one of the most widely used containerization systems because to its versatility and scalability. Additionally, it is considered as a significant element of an NFV architecture, the main enabling technology for NS [27].
- **Kubernetes**
  Kubernetes is a container orchestrator created by Google that contributed to the CNCF and is now open source. It benefits from Google’s years of experience in container management. It is a comprehensive solution for handling containerized application deployment, scheduling, and scaling, and it supports various containerization technologies, including Docker. Kubernetes-related services, support, and tools are widely available. It does not directly execute containers; rather, encapsulates one or more containers in a high-level architecture known as pods [28].
- **OpenStack**
  OpenStack is widely used for private and public cloud deployment by organizations of all sizes. Different solution suppliers implemented OpenStack for various 5G installations in the telecom sector. Over the last few years, OpenStack has been implemented on nine out of ten of the world’s largest telecom networks. It is also known as a cloud OS since it maintains and uses huge pools of resources in data centers, such as computing, networking, and storage [29].

## 4. Technical and Functional Realization of NS

**Figure 6:**
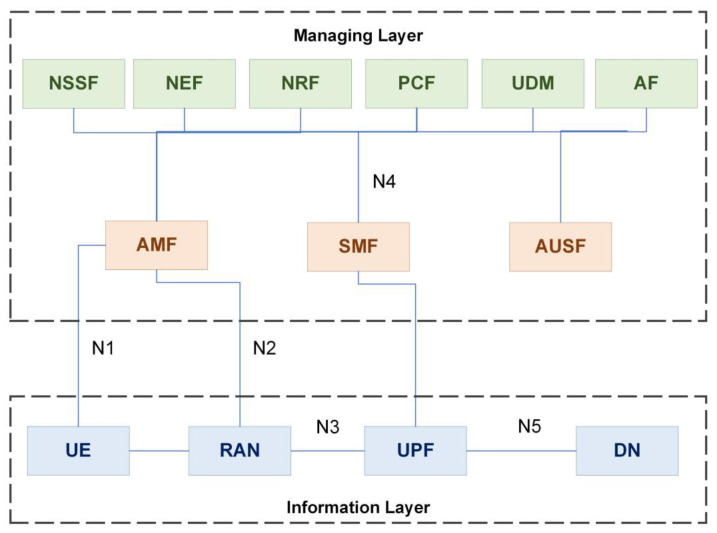
The 5G network core service-based architecture (SBA).

With a modular and cloud-native architecture, 5G significantly promises improvements to the CN. One major development is that it replaces conventional telecom design with service-based architecture (SBA), which encompasses all 5G activities such as authentication, safety, access control, and traffic aggregation from end devices. Briefly, SBA is a modeling approach indicated in Figure 6 that enables the autonomy and reliability of 5G network services. With minimal impact on other services, individual services may be upgraded directly and on-demand, enabling vendor flexibility, automation, and agile operating procedures, as well as faster delivery and deployment [30].

A summary of the 5G SBA network functions (NFs) includes:

- **Network Slice Selection Function (NSSF)**: appoints NSIs for the UE. Determines the AMF configured to service the UE.
- **Network Exposure Function (NEF)**: allows third-party programs to connect to the network in a secure manner.
- **Network Resource Function (NRF)**: ensures that records of services given by other NFs kept up to date.
- **Policy Control Function (PCF)**: to manage network performance, an integrated policy structure used. Control plane policy guidelines provided.
- **Unified Data Management (UDM)**: authentication and key agreement (AKA) credentials generated. Access granted depending on subscription data.
- **Application Function (AF)**: traffic routing choices, NEF accessibility, and strategic framework interactions are all managed through interfaces with the 3GPP core network.
- **Access and Mobility Management Function (AMF)**: licensing, permissions, and mobility management are all part of the AMF process.
- **Session Management Function (SMF):** Protocol data unit (PDU) sessions created, updated, or deleted.
- **Authentication Server Function (AUSF)**: authorization for 3GPP connection and non-3GPP connection that not trusted.
- **User Plane Function (UPF)**: redirecting and placement of user-plane packets. Mobility’s securing point.
- **Binding Support Function (BSF)**: connects an AF request to the appropriate PCF.

### 4.1. Concept of 5G SBA Registration

**Figure 7:**
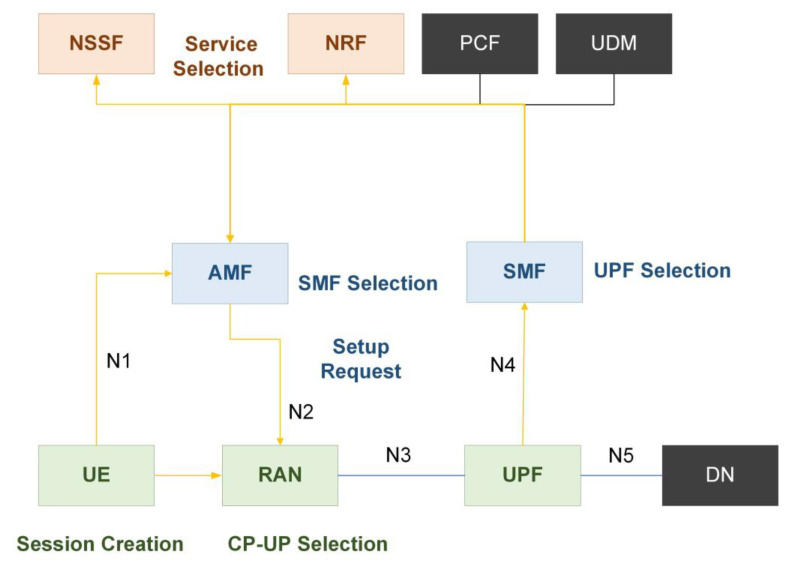
Registration logic of 5G SBA.

From a technical standpoint, a high-level Figure 7 used to implement the NS logic of how to pick or devote a particular combination of AMF, SMF, UPF, and a portion of radio to a certain subscriber. During registration, UE provides the desired NSSI to help the network assign the appropriate slice to the UE. NSSI’s structure comprises slice service type and slice description, which specify which slice it belongs to. As a result, the NSSI factor may be employed in RAN selection to determine which AMF should serve to that UE. If the RAN were able to send the message to the AMF, and the AMF received the UE’s registration request, it would proceed to the UDM and inquire about the subscribed NSSAI (S-NSSAIs) for this UE. If the AMF is unable to serve the requested slice, it will speak with the NSSF to determine which AMF shall provide this UE. Finally, it will arrive at PCF, where the network slice selection policy (NSSP) notifies the AMF as to where uplink traffic should direct. The call flow is completed at this point in the registration process by being able to allocate the AMF to the UE.

### 4.2. Concept of 5G SBA PDU Session 

**Figure 8:**
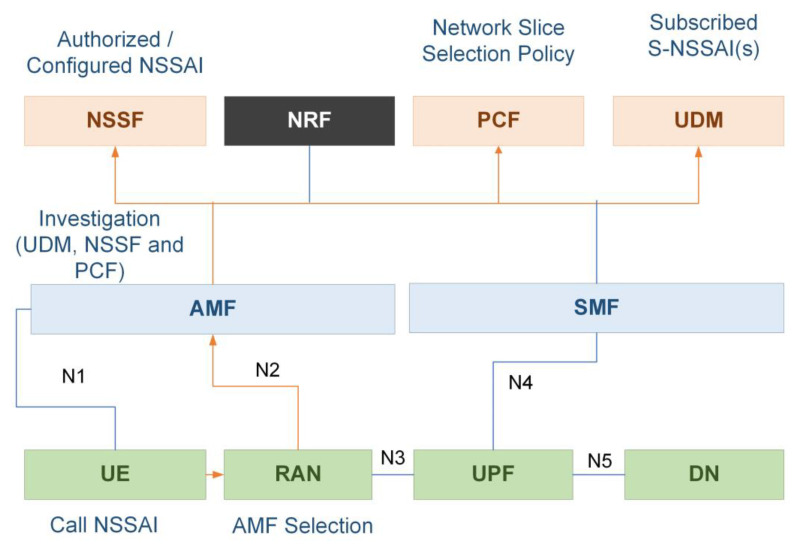
The 5G network SBA PDU session establishment.

The “PDU Session” concept for 5G user plane services, like the idea of a packet data network (PDN) connection in 4G EPC, is used to offer E2E user plane connectivity between the UE and a particular data network (DN). It is a post-registration procedure in which the UE sends a PDU session creation request to the AMF, which then selects the appropriate SMF for that UE as shown in Figure 8. The SMF resides on the allotted slice of a UE and will go to Nnssf to NSSF and request to pick the SMF. The NSSF will tell the AMF to send the slice indication to Nnrf, who will subsequently send the appropriate SMF to service the customer. After consulting the NRF, AMF may simply pick the SMF and then, using the same logic, it can go to the NRF and select the UPF based on the standard slice NSSI. So, it can notice that AMF, SMF, and UPF choices are all based on the slicing information [31].

### 4.3. Fifth-Generation NS Projects and Opportunities for Service Providers

This section provides a list of collaborative 5G NS research initiatives. With the fast evolution of 5G communication networks, vertical sectors are increasingly leveraging and implementing networking’s improved potential. Several 5GPPP NS initiatives are contributing to this approach by targeting the lifecycle of 5G vertical applications. As a result, current 5GPPP phase 2 and phase 3 initiatives have built a set of development, verification, and validation environments, with a primary focus on creating and designing vertical applications and virtual network capabilities. The initiatives relevant to NS listed in Table 5. 

## 5. Integrating Artificial Intelligence and Machine Learning for NS

Because NGWNs are diverse and dynamic, it is necessary to leverage powerful technologies to automate and enhance NS. Amazing advancements in AI research and applications have been made during the past decade. In recent times, ML, one of the most potent AI techniques, has rapidly advanced to include a wide range of applications, including speech recognition, picture processing, and self-driving automobiles. One important advantage of ML is its ability to manage complicated problems, which makes it a powerful tool that enhances the dynamic, diverse, and autonomous features of NGWN. By employing ML, large-scale systems may have advantages including improved performance, faster convergence, and performance optimization. Table 6 provides an overview of the AI and ML technology progress taking on various SDOs and industrial forums at the ETSI, ITU, ISO/IEC, and TM Forum.

## 6. Open Research Areas and Future Directions

With the introduction of NGWNs, the benefits of NS are well recognized; however, there are significant issues linked with this technology. The following are some undefined limitations that must be addressed.

- **RAN re-structuring and spectrum slicing**
  Microcells and macrocells must be redesigned to function in rationally defined slices. As the slices need to provide all access regardless of whether on Wi-Fi or on small cells or large-scale cells since appropriate co-appointment is required for the handover of sessions across slices. If a vehicle’s original equipment manufacturer (OEM) takes a slice, the administrator must allocate end-to-end network assets over every geographic district. Since the spectrum is additionally a mutual asset. This kind of committed provision is expensive. There is a need to develop a model for the enterprise customers that conveys the ideal QoE while still guaranteeing income for the administrator.
- **Service assurance and management**
  It is difficult to meet the SLAs established for each slice at each point in the system while using NS. If low latency is guaranteed, for example, the client should be able to get it. Similarly, while allocating slices, operators should ensure that unique slices adhere to the measuring system. Each slice must be scaled and monitored separately, and the slices must ensure QoS is fulfilled. Interoperability issues must be addressed when slices include a significant number of suppliers.
- **Cloud-native 5G core’s adaptability**
  The agility of the 5G core is essential to leverage cloud-native features such as automated processes, adaptive application and NF scaling, and greater utilization of storage and processing resources. The goal is to offer a completely compatible, scalable packet processing solution for containers.
- **Containers and VMs hybridization**
  Making NFs cloud-native might not be feasible since not all applications would benefit. The future may lie in the integration of the two technologies. To build an orchestration platform that can connect two distinct kinds of workloads, such as OpenStack and Kubernetes, more study is required.
- **Network slice isolation**
  The 5G mobile network is intended to enable the construction of network slices that might give access to third-party entities, such as companies. Developing an effective strategy to ensure isolation across various pods and services is a requirement for hosting extremely sensitive services in a network.
- **Dynamic service positioning**
  The 5G SBA is anticipated to comprise modular, dynamically connected NFs and services. Every service may offer a diverse set of features depending on the demands and service type. The self-contained smaller and more modular NFs joined and freely linked to instantiate an E2E network slice [55].
- **Administration trust among multiple regulatory domains**
  NS integration across various domains is a considerable challenge. In a situation with high mobility and the demand for uRLLC slicing, distributed slicing is necessary. The handling of security and trust across numerous vendors and several administrative domains that share physical resources must be investigated [56].
- **Edge intelligence and consumer estimation**
  The objective of NGWN is to make it possible for AI to employ wireless connectivity in scenarios such as self-learning networks and deep reinforcement learning (DRL). The idea of using the network edge wireless link is to develop effective and innovative neural network architectures [57].
- **Adaptive business mobility**
  The transfer of services used by a set of mobile users takes few seconds instead of the expected microseconds for the uRLLC slice’s E2E latency. When users relocate across the network, service migration might be costly, occupy limited bandwidth, and the target edge cloud may lack the resources needed to maintain the service continuity [58].
- **Efficient resource utilization using ML**
  ML methods, such as support vector machine (SVM) and DRL, are still to be managed efficiently in NS. SVM, for example, may use to aid in network slice selection by classifying service demands.
- **Controller placement solutions and isolation**
  The 5G core NFs may place in any geographical area across public, regional, or private DCs. The task is to determine the best assignment policy for the E2E network slice controller. The ideal number of controllers necessary is an outstanding issue that needs to study. Instead of a shared control plane, the issues are in providing an independent and tailored control plane for each customer. Consequently, the vertical business will be able to develop a tailored control programmed to satisfy the demands of its customers.

## 7. Conclusions

NS has proven to be critical for telecom carriers since it allows CSPs to provide only the services that each client needs. Many operators are eager to learn more about the technology and build a business structure around it. It is vital to implement the necessary restructuring and develop the necessary organizational mechanisms for exposing and capitalizing on such services in a timely and effective manner. We have presented an outline of slicing from a strategic viewpoint in terms of predictions and expected revenues in this paper. The requirement for an automation and orchestration framework for slicing acts as a catalyst for establishing the fundamental NS reinforcements in terms of actors, architectures, functions, and processes. Furthermore, we provide an outline of AI and ML-related initiatives taking place in various standards’ development groups and industrial forums. Finally, we highlighted certain unresolved challenges that require standardizing, such as cross-domain inter-working, SLA guarantee, intelligence, and automation.

## Figures and Tables

**Table 1:** Standardized SST values and their features.

SST	SST Value	Expected Features
eMBB	1	Extreme throughput Improved spectral efficiency Expanded coverage
uRLLC	2	High reliability Low latency High accessibility Place accuracy
mIoT	3	Higher linking density Fewer complication Prolonged coverage

**Table 2:** Summary of major surveys on NS and their objectives.

Studies	Applications	Classes	Business View	Orchestration	Cloud-Native Tools	Functional Realization	AI and ML	Challenges	Projects
[11]	✓	✕	✕	✓	✕	✕	✕	✓	✕
[12]	✓	✕	Limited	✓	✓	✓	✕	✓	✕
[13]	✓	✕	✕	✓	✕	✕	✕	✓	✕
[14]	✓	✕	Limited	✓	✕	✕	Limited	✓	✕
[15]	✓	✓	Limited	✕	Limited	✕	✓	✓	✓
Our Work	✓	✓	✓	✓	✓	✓	✓	✓	✓

**Table 3:** Summary of acronyms.

Acronyms	Full Form	Acronyms	Full Form	Acronyms	Full Form
NS	Network Slicing	uRLLC	Ultra-Reliable Low Latency Communication	VM	Virtual Machine
CSPs	Communication Service Providers	SST	Slice/Service Type	SBA	Services Based Architecture
AI	Artificial Intelligence	E2E	End-to-End	NFs	Network Functions
ML	Machine Learning	CAGR	Compound Annual Growth Rate	UE	User Equipment
NGWN	Next Generation Wireless Network	MNOs	Mobile Network Operators	NRF	Network Resource Function
MEC	Mobile Edge Computing	NSaaS	Network Slice as a Service	UDM	Unified Data Management
IoT	Internet of Things	CN	Core Network	AF	Application Function
3GPP	Third Generation Partnership Project	TN	Transport Network	AMF	Access and Mobility Management Function
SDO	Standard Developing Organization	RAN	Radio Access Network	SMF	Session Management Function
QoS	Quality of Service	CSI	Communication Service Instance	PDU	Protocol data unit
AR	Augmented Reality	NSSI	Network Slice Subnets Instances	UPF	User Plane Function
APN	Access Point Name	CSMF	Communication Service Management Function	AUSF	Authentication Server Function
EUTRAN	Evolved UMTS Terrestrial Radio Access Network	NSMF	Network Slice Management Function	BSF	Binding Support Function
EPC	Evolved Packet Core	NSSMF	Network Slice Subnet Management Function	PCF	Policy Control Function
DCN	Dedicated Core Network	NFMF	NF Management Function	DHCP	Dynamic Host Configuration Protocol
SDN	Software Defined Networking	VNF	Virtual Network Function	PDN	Packet Data Network
NFV	Network Function Virtualization	PNF	Physical Network Function	DN	Data Network
RAN	Radio Access Network	MANO	Management and Orchestration	CM	Configuration Management
SLA	Service Level Agreement	NFVO	NFV Orchestrator	FM	Fault Management
GSMA	Global System for Mobile Communications	NFVIs	NFV Infrastructure	ISG ENI	Industry Specification Group on Experiential Networked Intelligence
NSI	Network Slice Instance	LCM	Life Cycle Management	BPM	Business Process Management
ITU	International Telecommunication Union	VIM	Virtualized Infrastructure Manager	DRL	Deep Reinforcement Learning
eMBB	Enhanced Mobile Broadband	CNCF	Cloud-Native Core Network	SVM	Support Vector Machine

**Table 4:** Business requirements.

	Performance	Operational	Functional
**Business** **Constraints**	Throughput	Design and extensive process capacity	Isolation and flexibility
	Latency	SLAs and active preservation	Placement and delay tolerance
	Synchronization	Supervision potential	Security

**Table 5:** Summary of 5G NS initiatives.

Project Name	Application Areas	Tools SDN, NFV	Features	Objectives
5G-XHAUL (2015–2018) [32]	Automotive, e-health	✓,✓	NS concept and administration	Create a robust SDN control plane and request statistical models that are agility smart for optical/wireless 5G networks.
5G!PAGODA (2016–2019) [33]	IoT, human Interaction	✓,✓	Coherent architecture	The primary goals are to create a consistent infrastructure that allows Europe and Japan to collaborate on research and standards. The suggested innovations are designed to work with a common SDN/NFV-based architecture.
5G-MoNArch (2017–2019) [34]	Smart cities, industry 4.0	✓,✓	Software development and validation kits	Developed a detailed NS framework and used its flexibility to fully integrate functionalities necessary for industrial, media and entertainment, and smart city use cases.
ONE5G (2017–2019) [35]	Agricultural, automotive	✓,✓	NS design and management	To suggest enhanced network skills and modifications ahead of release fifteen to allow multi-service function and functional execution of “5G advanced (pro),” including upcoming network applications, advanced massive MIMO enablers, and link control.
SLICENET (2017–2020) [36]	Smart cities, e-health, smart grid	✓,✓	Software development and support	Generate a platform for smart network control, governance, and orchestration in SDN/NFV-enabled 5G networks to support infrastructure exchange across multiple operator domains.
5G-TANGO (2017–2020) [37]	Broadcasting, real-time comms, industry 4.0	✓,✓	_____	Provides commercial prospects through network adaptation and adaptation to vertical technical standards by decreasing the access barrier for third-party designers and enabling the building and integration of virtual network functions (VNFs) and application elements as “network services”.
MATILDA (2017–2019) [38]	Media, smart cities, automotive, industry 4.0	✓,✓	_____	Design a fundamental shift in the development of software for 5G-ready solutions, as well as virtual and physical network operations and network services. A cross virtualized infrastructure manager helps to manage cloud/edge computing and IoT resources from various locations.
5GCity (2017–2019) [39]	Smart cities, neutral masses, broadcasting	✓,✓	_____	Optimize the financial return for the whole virtual market chain and to deploy a common, multi-tenant, open forum that expands the (consolidated) cloud model to the network’s outer limit.
5G ESSENCE (2017–2020) [40]	Entertainment, public safety	✓,✓	_____	Manages the concepts of small cell as a service and edge cloud technology via enabling the drivers and reducing obstacles in the small cell industry, which anticipated to expand quickly and play a key role in the 5G ecosystem.
5G-TRANSFORMER (2017–2020) [41]	E-health, media, and entertainment, automotive	✓,✓	NS design and organization	Create a 5G network architecture centered on SDN/NFV that tailored to certain vertical sectors.
5GMobix (2018–2021) [42]	Associated and autonomous driving	✕,✕	Automated vehicle functionalities	Intends to link the benefits of 5G technology with sophisticated connected autonomous mobility applications to allow novel, traditionally implausible, autonomous car applications, both technically and commercially.
Primo-5G (2018–2021) [43]	Smart firefighting	✕,✕	Network framework	Demonstrate a comprehensive 5G system capable of providing interactive virtual solutions for moving items, achieved with cross-continental testbeds that connect radio access and core networks built by different project participants.
5G DRONES (2019–2022) [44]	eMBB, mIoT, uRLLC	✕,✕	Innovative developments	The drones intended to assess various UAV use-cases for eMBB, uRLLC, and mIoT 5G services as well as validate 5G KPIs for supporting them. The project will build on the ICT-17 projects’ 5G infrastructure and number of support locations while also identifying and developing the remaining elements.
INSPIRE-5Gplus (2019–2022) [45]	Self-directed and connected vehicles to critical industry 4.0	✕,✓	System framework, protection, and isolation	Intends to bring a significant shift in the access control of 5G networks and well beyond at the platform, vertical application, and quality of service.
AutoAir (2019) [46]	Authentication and advancement of associated and independent automobiles	✕,✕	System framework	Allow the testing and deployment of self—driving technology. In addition to requiring more network bandwidth than is currently available, fast travel speeds hinder cell-tower handoff. It will also look at whether these 5G connection options may be applied to both road and rail transportation.
MonB5G (2019–2022) [47]	Zero-touch processing and planning across business zones	✕,✕	E2E orchestration and protection	Allow NS at enormous sizes for 5G LTE and beyond, offer zero-touch administration and orchestration.
Semantic (2020–2023) [48]	Multi-GHz range networks, MEC-enabled use provisioning and E2E	✕,✕	E2E orchestration	Presents a unique research training system for multi-Ghz limit connectivity, MEC enabled approach encompasses, and E2E NS, all integrated and jointly managed with forward data service automatic control that powers the large amounts of portable BIG DATA triggered into the cellular connection.
Hexa-X (2021–2023) [49]	Sustainable growth, huge linking, tele-presence, and regional trust areas	✕,✓	System framework, scalability, protection, and orchestration	Aims to create leading technology enablers in the following areas: inherently new radio access techniques at high frequencies and resolution segmentation and sensor-based; integrated smartness via AI-driven radio interface and management for large scale deployments; 6G structural enablers for system partitioning and flexible reliability.

**Table 6:** The perspective of SDOs and industrial forums on using AI and ML.

Projects	Objectives
ETSI ISG ENI [50]	The Industry Specification Group on Experiential Networked Intelligence (ISG ENI) manages establishing policies that use AI mechanisms to improve the operator practical experience by identifying and combining evolving knowledge, allowing operators to make more prompt decisions, and aiding in network management and orchestration.
ITU FG-ML5G [51]	The goal of the ML5G Focus Group was to undertake an ML evaluation for future networks and to highlight significant gaps and concerns in standardized processes associated with this topic. In addition, technical elements such as use cases, requirements, and architectures are examined. The Focus Group operated as an open venue for specialists from ITU members and non-members to go forward with ML research linked to future networks, including 5G.
ISO/IEC JTC 1/SC42 [52]	Establish a set of standards for determining the context, resources, and processes for creating and deploying AI applications. It can be used by ISO, IEC, and JTC1 technical committees and subcommittees to expand on this work in developing standards for AI applications in their respective areas of interest. The recommendations give a high-level overview of the AI application environment, stakeholders and their responsibilities, the system’s life cycle, and common AI application features.
TM Forum Smart BPM [53,54]	SMART business process management (BPM) enables digital transformation catalyst who has previously proved the benefits of automated operations. This was accomplished through a business “process mining” and the instruments of adaptive discovery and orchestration of workflows. The use of analytics and big data also provided insights that enabled to leverage of user experience and network optimization.

## Data Availability

No data were used to support this study.

## References

[1] Iwamura M. (2015). NGMN view on 5G architecture. Proceedings of the 2015 IEEE 81st Vehicular Technology Conference (VTC Spring).

[2] Abdel Hakeem S.A., Hussein H.H., Kim H. (2022). Security Requirements and Challenges of 6G Technologies and Applications. Sensors.

[3] Zhang H., Liu N., Chu X., Long K., Aghvami A.-H., Leung V.C. (2017). Network slicing based 5G and future mobile networks: Mobility, resource management, and challenges. IEEE Commun. Mag..

[4] Elayoubi S.E., Jemaa S.B., Altman Z., Galindo-Serrano A. (2019). 5G RAN slicing for verticals: Enablers and challenges. IEEE Commun. Mag..

[5] Debbabi F., Jmal R., Fourati L.C., Ksentini A. (2020). Algorithmics and modeling aspects of network slicing in 5G and beyonds network: Survey. IEEE Access.

[6] Rost P., Banchs A., Berberana I., Breitbach M., Doll M., Droste H., Mannweiler C., Puente M.A., Samdanis K., Sayadi B. (2016). Mobile network architecture evolution toward 5G. IEEE Commun. Mag..

[7] Erunkulu O.O., Zungeru A.M., Lebekwe C.K., Mosalaosi M., Chuma J.M. (2021). 5G mobile communication applications: A survey and comparison of use cases. IEEE Access.

[8] Chahbar M., Diaz G., Dandoush A., Cérin C., Ghoumid K. (2020). A comprehensive survey on the E2E 5G network slicing model. IEEE Trans. Netw. Serv. Manag..

[9] Zhou X., Li R., Chen T., Zhang H. (2016). Network slicing as a service: Enabling enterprises’ own software-defined cellular networks. IEEE Commun. Mag..

[10] Li Q., Wu G., Papathanassiou A., Mukherjee U. (2016). An end-to-end network slicing framework for 5G wireless communication systems. arXiv.

[11] Foukas X., Patounas G., Elmokashfi A., Marina M.K. (2017). Network slicing in 5G: Survey and challenges. IEEE Commun. Mag..

[12] Afolabi I., Taleb T., Samdanis K., Ksentini A., Flinck H. (2018). Network slicing and softwarization: A survey on principles, enabling technologies, and solutions. IEEE Commun. Surv. Tutor..

[13] Zhang S. (2019). An overview of network slicing for 5G. IEEE Wirel. Commun..

[14] Khan L.U., Yaqoob I., Tran N.H., Han Z., Hong C.S. (2020). Network slicing: Recent advances, taxonomy, requirements, and open research challenges. IEEE Access.

[15] Wijethilaka S., Liyanage M. (2021). Survey on network slicing for Internet of Things realization in 5G networks. IEEE Commun. Surv. Tutor..

[16] Chin W.H., Fan Z., Haines R. (2014). Emerging technologies and research challenges for 5G wireless networks. IEEE Wirel. Commun..

[17] Khan A.A., Abolhasan M., Ni W., Lipman J., Jamalipour A. (2021). An end-to-end (E2E) network slicing framework for 5G vehicular ad-hoc networks. IEEE Trans. Veh. Technol..

[18] Walia J.S., Hämmäinen H., Kilkki K., Flinck H., Yrjölä S., Matinmikko-Blue M. (2021). A virtualization infrastructure cost model for 5g network slice provisioning in a smart factory. J. Sens. Actuator Netw..

[19] Taleb T., Afolabi I., Bagaa M. (2019). Orchestrating 5G network slices to support industrial internet and to shape next-generation smart factories. IEEE Netw..

[20] Rehman A.U., Naqvi R.A., Rehman A., Paul A., Sadiq M.T., Hussain D. (2020). A trustworthy siot aware mechanism as an enabler for citizen services in smart cities. Electronics.

[21] Abbas K., Khan T.A., Afaq M., Song W.-C. (2021). Network slice lifecycle management for 5g mobile networks: An intent-based networking approach. IEEE Access.

[22] Muruganathan S.D., Lin X., Määttänen H.-L., Sedin J., Zou Z., Hapsari W.A., Yasukawa S. (2021). An overview of 3GPP release-15 study on enhanced LTE support for connected drones. IEEE Commun. Stand. Mag..

[23] Habibi M.A., Han B., Nasimi M., Kuruvatti N.P., Fellan A., Schotten H.D. (2021). Towards a fully virtualized, cloudified, and slicing-aware RAN for 6G mobile networks. 6G Mobile Wireless Networks.

[24] Taleb T., Afolabi I., Samdanis K., Yousaf F.Z. (2019). On multi-domain network slicing orchestration architecture and federated resource control. IEEE Netw..

[25] Sciancalepore V., Mannweiler C., Yousaf F.Z., Serrano P., Gramaglia M., Bradford J., Pavón I.L. (2019). A future-proof architecture for management and orchestration of multi-domain NextGen networks. IEEE Access.

[26] Nguyen T.T., Pham T.M. (2020). Efficient Traffic Engineering in an NFV Enabled IoT System. Sensors.

[27] Addad R.A., Taleb T., Flinck H., Bagaa M., Dutra D. (2020). Network slice mobility in next generation mobile systems: Challenges and potential solutions. IEEE Netw..

[28] Casalicchio E. (2019). Container orchestration: A survey. Systems Modeling: Methodologies and Tools.

[29] Hao J., Ye K., Xu C.-Z. (2019). Live migration of virtual machines in OpenStack: A perspective from reliability evaluation. Proceedings of the International Conference on Cloud Computing.

[30] Prashant S., Abeer A., Sabih R., Nabil G., Imran M., Samrah A. (2021). Network slicing: A next generation 5G perspective. EURASIP J. Wirel. Commun. Netw..

[31] Shah S.D.A., Gregory M.A., Li S. (2021). Cloud-native network slicing using software defined networking based multi-access edge computing: A survey. IEEE Access.

[32] Camps-Mur D., Gutierrez J., Grass E., Tzanakaki A., Flegkas P., Choumas K., Giatsios D., Beldachi A.F., Diallo T., Zou J. (2019). 5G-XHaul: A novel wireless-optical SDN transport network to support joint 5G backhaul and fronthaul services. IEEE Commun. Mag..

[33] Afolabi I., Ksentini A., Bagaa M., Taleb T., Corici M., Nakao A. (2017). Towards 5G network slicing over multiple domains. IEICE Trans. Commun..

[34] Gutierrez-Estevez D.M., Dipietro N., Dedomenico A., Gramaglia M., Elzur U., Wang Y. (2018). 5G-MoNArch use case for ETSI ENI: Elastic resource management and orchestration. Proceedings of the 2018 IEEE Conference on Standards for Communications and Networking (CSCN).

[35] Schaich F., Hamon M.-H., Hunukumbure M., Lorca J., Pedersen K., Schubert M., Kosmatos E., Wunder G., Reaz K. (2018). The ONE5G approach towards the challenges of multi-service operation in 5G systems. Proceedings of the 2018 IEEE 87th Vehicular Technology Conference (VTC Spring).

[36] Wang Q., Alcaraz-Calero J., Weiss M.B., Gavras A., Neves P.M., Cale R., Bernini G., Carrozzo G., Ciulli N., Celozzi G. (2018). SliceNet: End-to-end cognitive network slicing and slice management framework in virtualised multi-domain, multi-tenant 5G networks. Proceedings of the 2018 IEEE international symposium on broadband multimedia systems and broadcasting (BMSB).

[37] Soenen T., Tavernier W., Peuster M., Vicens F., Xilouris G., Kolometsos S., Kourtis M.-A., Colle D. (2019). Empowering network service developers: Enhanced nfv devops and programmable mano. IEEE Commun. Mag..

[38] Bruschi R., Pajo J.F., Davoli F., Lombardo C. (2021). Managing 5G network slicing and edge computing with the MATILDA telecom layer platform. Comput. Netw..

[39] Marabissi D., Mucchi L., Fantacci R., Spada M.R., Massimiani F., Fratini A., Cau G., Yunpeng J., Fedele L. (2019). A Real Case of Implementation of the Future 5G City. Future Internet.

[40] Spada M.R., Pérez-Romero J., Sanchoyerto A., Solozabal R., Kourtis M.-A., Riccobene V. (2019). Management of mission critical public safety applications: The 5G ESSENCE project. Proceedings of the 2019 European Conference on Networks and Communications (EuCNC).

[41] Li X., Garcia-Saavedra A., Costa-Perez X., Bernardos C.J., Guimarães C., Antevski K., Mangues-Bafalluy J., Baranda J., Zeydan E., Corujo D. (2021). 5Growth: An end-to-end service platform for automated deployment and management of vertical services over 5G networks. IEEE Commun. Mag..

[42] Serrador A., Mendes C., Datia N., Cota N., Cruz N., Beire A.R. (2021). A performance measurement platform for C-ITS over 5G. Proceedings of the 2021 Joint European Conference on Networks and Communications & 6G Summit (EuCNC/6G Summit).

[43] Sung K.W., Mutafungwa E., Jäntti R., Choi M., Jeon J., Kim D., Kim J., Costa-Requena J., Nordlöw A., Sharma S. (2019). PriMO-5G: Making firefighting smarter with immersive videos through 5G. Proceedings of the 2019 IEEE 2nd 5G World Forum (5GWF).

[44] Si-Mohammed S., Bouaziz M., Hellaoui H., Bekkouche O., Ksentini A., Taleb T., Tomaszewski L., Lutz T., Srinivasan G., Jarvet T. (2020). Supporting unmanned aerial vehicle services in 5G networks: New high-level architecture integrating 5G with U-space. IEEE Veh. Technol. Mag..

[45] Ortiz J., Sanchez-Iborra R., Bernabe J.B., Skarmeta A., Benzaid C., Taleb T., Alemany P., Muñoz R., Vilalta R., Gaber C. INSPIRE-5Gplus: Intelligent security and pervasive trust for 5G and beyond networks. Proceedings of the Proceedings of the 15th International Conference on Availability, Reliability and Security.

[46] Wilson P. (2019). State of smart cities in UK and beyond. IET Smart Cities.

[47] Kukliński S., Kołakowski R., Tomaszewski L., Sanabria-Russo L., Verikoukis C., Phan C.-T., Zanzi L., Devoti F., Ksentini A., Tselios C. (2021). MonB5G: AI/ML-Capable Distributed Orchestration and Management Framework for Network Slices. Proceedings of the 2021 IEEE International Mediterranean Conference on Communications and Networking (MeditCom).

[48] Sana M., Strinati E.C. (2022). Learning Semantics: An Opportunity for Effective 6G Communications. Proceedings of the 2022 IEEE 19th Annual Consumer Communications & Networking Conference (CCNC).

[49] Uusitalo M.A., Ericson M., Richerzhagen B., Soykan E.U., Rugeland P., Fettweis G., Sabella D., Wikström G., Boldi M., Hamon M.-H. Hexa-X The European 6G flagship project. Proceedings of the 2021 Joint European Conference on Networks and Communications & 6G Summit (EuCNC/6G Summit).

[50] Wang Y., Forbes R., Elzur U., Strassner J., Gamelas A., Wang H., Liu S., Pesando L., Yuan X., Cai S. (2020). From design to practice: ETSI ENI reference architecture and instantiation for network management and orchestration using artificial intelligence. IEEE Commun. Stand. Mag..

[51] Kafle V.P., Hirayama T., Miyazawa T., Jibiki M., Harai H. (2021). Network Control and Management Automation: Architecture Standardization Perspective. IEEE Commun. Stand. Mag..

[52] Kafle V.P., Fukushima Y., Martinez-Julia P., Miyazawa T. (2018). Consideration on automation of 5G network slicing with machine learning. Proceedings of the 2018 ITU Kaleidoscope: Machine Learning for a 5G Future (ITU K).

[53] Yigitcanlar T., Desouza K.C., Butler L., Roozkhosh F. (2020). Contributions and risks of artificial intelligence (AI) in building smarter cities: Insights from a systematic review of the literature. Energies.

[54] Fernandes C., Cruz R.A. (2021). Business processes model for the integration of over-the-top platforms in communications service providers operations. J. Inf. Syst. Eng. Manag..

[55] Zhou J., Zhao W., Chen S. (2020). Dynamic network slice scaling assisted by prediction in 5G network. IEEE Access.

[56] Drif Y., Chaput E., Lavinal E., Berthou P., Tiomela Jou B., Gremillet O., Arnal F. (2021). An extensible network slicing framework for satellite integration into 5G. Int. J. Satell. Commun. Netw..

[57] Katz M., Matinmikko-Blue M., Latva-Aho M. (2018). 6Genesis flagship program: Building the bridges towards 6G-enabled wireless smart society and ecosystem. Proceedings of the 2018 IEEE 10th Latin-American Conference on Communications (LATINCOM).

[58] Parvez I., Rahmati A., Guvenc I., Sarwat A.I., Dai H. (2018). A survey on low latency towards 5G: RAN, core network and caching solutions. IEEE Commun. Surv. Tutor..

